# The NIA LINKAGE Enclave Application Process

**DOI:** 10.1093/geroni/igaf122.575

**Published:** 2025-12-31

**Authors:** Marcela Blinka, Jennifer Albrecht, Adam Spira, Halima Amjad, Alden Gross, Emerson M Wickwire, Atul Malhotra, Christopher Kaufmann

**Affiliations:** Johns Hopkins University, Center on Aging and Health, Baltimore, Maryland, United States; University of Maryland, Baltimore, Maryland, United States; Johns Hopkins Bloomberg School of Public Health, Baltimore, Maryland, United States; Johns Hopkins University School of Medicine, Baltimore, Maryland, United States; Johns Hopkins University School of Medicine, Baltimore, Maryland, United States; University of Maryland, Baltimore, Maryland, United States; University of California, San Diego, San Diego, California, United States; University of Florida, Gainesville, Florida, United States

## Abstract

As the global population continues to age, aging-related diseases, including Alzheimer’s disease and other dementias, are becoming more common, creating serious challenges for healthcare systems worldwide. Addressing these challenges requires a comprehensive understanding of the genetic, environmental, and behavioral factors that contribute to the development and progression of these conditions. The National Institute on Aging Data LINKAGE Program (LINKAGE) is a valuable resource, providing access to a wide range of data on genetic variations, biomarkers, and age-related diseases with secure linkages to administrative claims data. Researchers can apply to LINKAGE to gain access to these data, which are essential for improving our understanding of these conditions and advancing efforts in diagnosis, prevention, and treatment. In this presentation, we will share our experience in applying for access to these data through the LINKAGE program, with a particular focus on the National Health and Aging Trends Study (NHATS) and the Health and Retirement Study (HRS). We will highlight the lessons learned throughout the process and offer practical recommendations for navigating the application system more effectively. Based on our experience, we found that engaging with NHATS and HRS personnel early and often throughout the application process is critical to success. Additionally, close coordination between study partners significantly streamlines the process, ensuring smoother communication and faster access to valuable data. By sharing these insights, we aim to help other researchers expedite their own applications and make the most of the resources available through LINKAGE.

